# A systematic review of delay in the diagnosis and treatment of tuberculosis

**DOI:** 10.1186/1471-2458-8-15

**Published:** 2008-01-14

**Authors:** Dag Gundersen Storla, Solomon Yimer, Gunnar Aksel Bjune

**Affiliations:** 1 Department of International Health, Institute of General Practice and Community Medicine, University of Oslo, PO Box 1130 Blindern, N-0318 Oslo, Norway; 2 Competence Centre for Imported and Tropical Diseases, Ullevål University Hospital, Oslo, Norway

## Abstract

**Background:**

Early diagnosis and immediate initiation of treatment are essential for an effective tuberculosis (TB) control program. Delay in diagnosis is significant to both disease prognosis at the individual level and transmission within the community. Most transmissions occur between the onset of cough and initiation of treatment.

**Methods:**

A systematic review of 58 studies addressing delay in diagnosis and treatment of TB was performed. We found different definitions of, for example, debut of symptoms, first appropriate health care provider, time to diagnosis, and start of treatment. Rather than excluding studies that failed to meet strict scientific criteria (like in a meta-analysis), we tried to extract the "solid findings" from all of them to arrive on a more global understanding of diagnostic delay in TB.

**Results:**

The main factors associated with diagnostic delay included human immunodeficiency virus; coexistence of chronic cough and/or other lung diseases; negative sputum smear; extrapulmonary TB; rural residence; low access (geographical or sociopsychological barriers); initial visitation of a government low-level healthcare facility, private practitioner, or traditional healer; old age; poverty; female sex; alcoholism and substance abuse; history of immigration; low educational level; low awareness of TB; incomprehensive beliefs; self-treatment; and stigma.

**Conclusion:**

The core problem in delay of diagnosis and treatment seemed to be a vicious cycle of repeated visits at the same healthcare level, resulting in nonspecific antibiotic treatment and failure to access specialized TB services. Once generation of a specific diagnosis was in reach, TB treatment was initiated within a reasonable period of time.

## Background

Until the last part of the twentieth century, tuberculosis (TB) was a major cause of death in both developed and developing countries. Due to a range of factors such as the human immunodeficiency virus (HIV) epidemic, population growth, migration, socioeconomic changes, and broad spread of aggressive and resistant new strains such as the Beijing and W strains, a resurgence of TB has occurred, even in low endemic areas [1]. In 1993, the World Health Organization (WHO) declared a state of global emergency for TB due to the steady worldwide increase in the disease. Along with HIV and malaria, TB has been declared a global enemy. In 2005, 12 million new cases of tuberculosis were identified, a 58% increase from the 7.5 million estimated cases in 1990, and it was estimated that in 2005 the disease caused 1.5 million deaths worldwide [2].

The goal of TB control programs is to arrest transmission within the community. Achieving this goal takes considerable time, because most individuals in endemic areas are already infected, constituting a reservoir that continuously contributes to the pool of infectious cases. An effective TB control program requires early diagnosis and immediate initiation of treatment. Delay in diagnosis is significant with regard to not only disease prognosis at the individual level but also transmission within the community and the reproductive rate of the TB epidemic [3,4]. Most transmissions occur between the appearance of cough and initiation of treatment. Madebo et al found that patients become more contagious as the delay progresses; the longest delays are associated with the highest bacillary numbers on sputum smears [5]. Because TB symptoms, particularly chronic cough with sputum, are so prevalent in most societies, early contact with health services causes delay rather than suspicion of TB. We found that in Ethiopia patients with TB symptoms contact an educated health worker on average after just 25% of the total delay period [6]. Thus, there is a fourfold-difference in the time of first contact and diagnosis.

Analysis of the factors leading to this delay between first contact and diagnosis is crucial to combatting the increasing TB epidemic. Although there are multiple studies of delayed TB diagnosis, no one has performed a systematic review.

## Methods

### Search strategy

We searched the following databases using the search terms and strategy described in Table 1: the Cochrane Infectious Diseases Group Specialized Register (February 2007); the Cochrane Central Register of Controlled Trials (CENTRAL) published in The Cochrane Library (February 2007); MEDLINE (1966 to February 2007); EMBASE (1974 to February 2007); and LILACS (1982 to February 2007). In addition, to identify unpublished and ongoing studies, we contacted individual researchers in the TB field as well as the WHO (2006) and the International Union Against Tuberculosis and Lung Disease (IUATLD, 2006).

**Table 1:** Search terms and strategy

**Search set**	**CIDG SR**	**CENTRAL**	**MEDLINE**	**EMBASE**	**LILACS**
1	Tuberculosis	Tuberculosis	Tuberculosis	Tuberculosis	Tuberculosis
2	Diagnostic delay	Diagnostic delay	Diagnostic delay	Diagnostic delay	Demora diagnostico*
3		Treatment delay	Treatment delay	Treatment delay	
4		Treatment seeking	Treatment seeking	Treatment seeking	
5		Case finding	Case finding	Case finding	
6		Help seeking	Help seeking	Help seeking	

CIDG SR: Cochrane Infectious Diseases Group Specialized Register

CENTRAL: Cochrane Central Register of Controlled Trials

*Demora diagnostico: Diagnostic delay

### Selection and analysis

Only observational studies were selected. All obtainable studies of patients receiving treatment for active pulmonary TB that recorded at least the median or mean total delay in diagnosis were included. The outcomes of interest were diagnostic delay from the debut of symptoms to the time of diagnosis or start of treatment. The titles and abstracts of the identified reports were used to exclude studies that clearly did not meet the inclusion criteria. For studies deemed potentially eligible for inclusion, we obtained the full paper. We screened the full articles of selected studies to confirm eligibility and resolved any disagreements by discussion. Our intent was not to exclude studies based on strict scientific criteria, or to perform a traditional quality assessment, but to make the studies as comparable as possible.

We analyzed the studies with the intent of identifying differences in approaches, rather than to define a gold standard. A primary aim was to describe the inevitable inaccuracy that arises from the use of different definitions of, for example, the debut of symptoms, first appropriate health care provider, time of diagnosis, and start of treatment.

## Results

### Search results

Our analysis revealed how complex it is to define diagnostic delay, and there were major differences between studies regarding inclusion and exclusion criteria, onset of symptoms, first contact and end of delay. First, the 58 studies used different inclusion criteria. Seventeen studies included all new TB cases, 11 included all pulmonary TB cases, 3 included all cases with a positive sputum smear, 24 included all new cases with a positive sputum spear, and for 3 studies data were not obtainable. Likewise, the study exclusion criteria differed. Some studies carefully excluded all cases with chronic underlying pulmonary conditions that could interfere with the patient's definition of symptom onset, but most did not. Some studies excluded visitors, mortal cases, and individuals with mental disturbances. The age-related exclusion criteria also varied: most studies excluded cases below the age of 16 years, some excluded cases below the age of 18 years, and a few included children of all ages. One study did not include patients who had undergone 2 or more months of treatment.

Definition of the onset of symptoms was also variable. Forty-nine studies defined onset as the debut of any symptom, 2 studies defined onset as debut of cough, and 1 study defined onset as debut of any pulmonary symptom. For 6 studies, a definition of symptom onset could not be obtained.

With regard to definition of the first contact, 34 studies defined the first contact as the first visit to a qualified healthcare provider. However, some of these studies included any allopathic ("western medicine") provider within the category of a qualified healthcare provider; others used the time of first contact with the national TB program in defining the end of patient delay. Eighteen studies defined the first contact as the time when the patient sought contact with any healthcare provider outside the household, including traditional practitioners. Four studies recorded both. Six studies did not provide any information with regard to definition of the first contact.

The studies also applied different definitions of the end of the delay. Seventeen studies defined the end of healthcare system delay as the time when a correct diagnosis was made (diagnostic delay), 20 studies defined it as the time the patient started treatment (treatment delay), and 14 studies distinctly recorded both. Data of this kind were unavailable for 7 studies.

Most studies defined the delay as a specific number of days, but several studies defined it as greater than a specific period of time (e.g. >60 days, > 90 days), or delay was defined as significantly longer in one group versus another group. A cut off point of 30 days to dichotomize into either delay on non delay was also commonly used.

### Diagnostic delay

Table 2 lists the included studies in descending order according to the total diagnostic delay. Not surprisingly, the longest total delays (> 120 days) were reported for some high endemic countries, with the exception of the median 126-day delay reported by Lewis et al for East London [7]. Most of the studies, whether investigating low or high endemic countries, reported a total diagnostic delay within the range of 60–90 days (mean ± standard deviation: 72 days ± 28 days).

**Table 2:** Median diagnostic delay for pulmonary TB patients in 58 studies

**Country**	**Year**	**First author**	**DD Pat**	**DD HCP**	**DD total**	**Ref no.**
Tanzania	2000	Wandwalo	15	120	136	[29]
United Kingdom	2003	Lewis	63	35	126	[7]
Burkina Faso	2006	Ouedraogo	*	*	120	[49]
Ethiopia	1999	Madebo	*	*	120	[5]
Ghana	1998	Lawn	28	56	120	[11]
Malawi	1988	Nkhoma	*	*	120	[54]
Thailand	1991	Tesana	*	*	120	[55]
Thailand	1993	Pungrassami	*	*	120	[45]
Romania	1989	Anastasatu	69♠	34♠	107♠	[33]
Iran	2002	Masjedi	13♠		93♠	[51]
Vietnam	1999	Long	54♠	29♠	93♠	[30]
Pakistan	2006	WHO	9	87	91	[27]
Malaysia	1994	Hooi	15	35	90	[21]
United States	2005	Golub	32	26	89	[22]
Iran	2006	WHO	24	42	88	[27]
Malaysia	1997	Liam	14	49	88	[36]
Botswana	1998	Steen	21	35	84	[26]
New Zealand	2000	Calder	7	49	84	[44]
Uganda	2005	Kiwuwa	14	63	84	[23]
Spain	2003	Altet Gomez	43♠	39♠	82♠	[24]
Ethiopia	2005	Yimer	15	61	80	[6]
Nepal	2001	Yamasaki-Nakagawa	23	29	79	[14]
Japan	1990	Niijima	*	*	78†	[46]
Mongolia	1996	Enkhbat	29	35	78	[35]
Nigeria	2004	Odusanya	56	7	70	[19]
South Africa	2001	Pronyk	28	7	70	[8]
Australia	2001	Ward	30	11	66	[20]
Thailand	2006	Rojpibulstit	31	20	66	[9]
China	2004	Bai	30	24	65	[28]
Italy	2006	Gagliotti	7	36	65	[38]
Spain	1996	Franco	23	32	64	[12]
Turkey	2004	Güneylioglu	18♠	13♠	64♠	[16]
Ethiopia	2002	Demissie	60	6	64	[18]
Norway	2006	Farah	28	33	63	[17]
United States	1998	Asch			60▲	[42]
India	2002	Rajeswari	20	23	60	[25]
Korea	1992	Mori	54♠	14♠	60♠	[31]
Peru	1996	Chavez	*	*	60	[53]
The Gambia	2001	Lienhardt	*	*	60	[32]
Zambia	2001	Needham	*	*	60	[10]
Cambodia	2006	Saly	*	10	58	[50]
Somalia	2006	WHO	53	7	58	[27]
Malawi	2000	Salaniponi	*	*	56	[47]
Syria	2006	WHO	31	15	55	[27]
China	2005	Xu	10	39	50	[37]
Turkey	2006	Okur	30	19	49	[34]
United Kingdom	2007	Rodger	*	*	49	[40]
Australia	1996	Pirkis	*	*	44	[15]
Taiwan	2005	Chiang	7	23	44	[56]
Egypt	2004	WHO	12	18	42	[27]
Japan	2002	Sasaki	21	7	42	[52]
Iraq	2004	WHO	31	2	36	[27]
United States	1999	Sherman	21	6	35	[39]
Yemen	2004	WHO	28	4	35	[27]
China	2006	Deng	19	5	31	[41]
France	1996	Gulbaran			30♠	[48]
Thailand	2001	Ngamvithayapong	11	8	*	[13]
Pakistan	2001	Sadiq			21€	[57]

The studies are listed in descending order of the median diagnostic delay.

DD Pat (Diagnostic Delay by the Patients): Time from debut of symptoms to first visit to health care provider

DD HCP (Diagnostic Delay by the Health Care Providers): Time from first visit to a HCP to the making of a proper diagnosis

DD Total (Total Diagnostic Delay): Time from debut of symptoms to the making of a proper diagnosis

* Data not obtainable

† Average calculated by the reviewers from separate numbers for female and male

♠ Mean

▲ 80% percentile

€ 77% percentile

There was no consistent pattern with regard to the relative contributions of patients and healthcare providers to the diagnostic delay. The main delay was patient related in the studies in London (Lewis et al), Romania, Vietnam, Nigeria, South Africa, Australia (Queensland), Ethiopia (Addis Abeba), Korea, Somalia, Syria, Turkey (Istanbul, Okur et al), Japan (Chiba), Iraq, USA (New York), Yemen, and China (Shanghai). The main cause of delay was identified as the healthcare system in the studies of Tanzania, Ghana, Pakistan, Malaysia, Iran (WHO, nationwide), Botswana, New Zealand, Uganda, Ethiopia (Amhara), Italy, and China (Jianhu). Twelve studies reported a nearly equal contribution of patients and healthcare system to the diagnostic delay. The remaining studies did not record the relative importance of these two factors in the diagnostic delay.

### Symptoms prior to diagnosis

Twenty-five studies recorded the frequency of symptoms reported by patients before diagnosis. The average frequencies of the following five cardinal symptoms were (number of studies listing the symptom in brackets): cough 85% (25), fever 65% (24), weight loss 62% (22), chest symptoms 50% (24), and haemoptysis 25% (22). Other symptoms less frequently reported: sputum 67% (5), fatigue 55% (8), and increased sweating 35% (10). All but two studies defined the onset of patient delay from the debut of any symptom [8,9], where the debut of cough defined the start.

### Risk factors for prolonged diagnostic delay

The possible risk factors for diagnostic delay were heterogeneous (Table 3). The study conclusions were also heterogeneous; a risk factor for increased delay in some studies was a risk factor for decreased delay in other studies. Some factors were identified in numerous studies, while others were mentioned by only one study or a few studies. Following is a brief analysis of the factors, which are further elaborated in the "Discussion".

**Table 3:** Risk factors for diagnostic delay

**Risk factor**	**Positive association with risk**	**Negative association with risk**
HIV	[10]	[11-13]
Coexistence of chronic cough and/or other lung diseases	[12, 14-16]	[7]
Negative sputum smear	[12, 19, 20]	[15]
Extrapulmonary TB	[7, 17, 18]	
Rural residence	[5, 11, 14, 16, 23, 25, 29-32]	
Low access to healthcare (geographical or socio-psychological barriers)	[6, 8, 10, 14, 18, 23, 25, 27-30, 34, 42, 47, 48, 50]	
Initial visit to government low-level healthcare facility	[5, 6, 9-11, 23, 26, 32-34]	[35]
Initial visit to traditional or unqualified practitioner	[9, 10, 14, 26-29, 32, 36, 37]	
Initial visit to private practitioner	[9, 10, 14, 26-29, 32, 36, 37]	
Initial visit to tertiary-level services/hospital	[11]	[13, 23, 38, 39]
Old age	[5, 12, 14-16, 19, 23, 24, 26, 38, 40, 41]	[18, 35]
Poverty	[7, 20, 21, 27, 28, 34, 37, 40, 41, 47, 48, 54, 56]	[18]
Female sex	[8, 10, 11, 14-16, 20, 22, 31, 33, 39, 40]	[5, 21, 23, 25]
Alcoholism or substance abuse	[8, 21-25]	
History of immigration	[8, 15, 17, 22, 38, 39, 42]	
Low educational level and/or low awareness and knowledge about TB	[9, 15-17, 20, 21, 23, 24, 27, 28, 31-33, 38, 39]	[13] (low educational level)
Other	Health-related reasons: Generally poor health [26] Smoking [14, 23] Coexistence of sexually transmitted diseases [26] Less severe and indifferent symptoms [27] No haemoptysis [16, 28]	
	Socioeconomic factors: Married [5] Single [18, 26] Large family size [30] Farmer [5] White (vs. aboriginal) [20] Muslim [18] Belonging to an indigenous group [13] No insurance [13]	
	Beliefs and attitudes: Beliefs about TB (not curable, caused by evil spirits, etc.) [8, 14, 27] Stigma [27] Self-treatment [6, 36, 42]	

The columns are identifying the applicable studies finding positive and negative association, respectively, with the risk factors

#### Clinical characteristics

The WHO study in Syria found HIV to be a risk factor for increased delay in diagnosis [10], while three other studies concluded the opposite [11-13]. Four studies found coexistence of chronic cough and/or other lung diseases be a risk factor for increased delay [12,14-16]; one study found the opposite [7]. Only a few studies included extrapulmonary TB, and as expected three found that patients with extrapulmonary TB experience longer delays than do patients with pulmonary TB [7,17,18]. Three studies found a negative sputum smear to be a risk factor for increased delay [12,19,20]; one study found the opposite (the WHO study in Egypt [15]). Six studies found alcoholism or substance abuse to be a risk factor for increased diagnostic delay [8,21-25]. Other health-related risk factors identified include generally poor health [26], smoking [14,23], coexistence of sexually transmitted diseases [26], less severe and indifferent symptoms [27], and absence of hemoptysis [16,28].

#### Socioeconomic factors

A range of studies found rural residence to be a risk factor for prolonged delay [5,11,14,16,23,25,29-32]. This risk factor seems to be closely linked to low access to healthcare and choice of settings in which to first seek healthcare (see next section). Even among developing countries, access to healthcare varied. For example, in Ethiopia the public health service coverage was reported to be 50% [6], whereas in The Gambia 87% of the population was reported to have good access to healthcare [7,19,20,32]. The studies provided broad evidence that low access leads to prolonged delay in diagnosis [5,7,9,13,17,20,27-33].

#### Sociopsychological factors

Seeking government low-level health care facility first [5,6,9-11,23,26,32-34] (one study from Spain found the opposite [35]). Initially seeking a traditional or unqualified practitioner [9,10,14,26-29,32,36,37]. First seeking a private practitioner was a clear risk factor for diagnostic delay, independent of rural or urban residence [9-11,14,15,19,21,23,24,26,28,35]. Four studies concluded that seeking specialized services leads to a decreased diagnostic delay [13,23,38,39], while one study from the USA [11] found the opposite.

#### Sociodemographic factors

A range of studies found old age to be a risk factor for increased diagnostic delay [6,13,15-17,20,23,25,32,37-39], while two studies found the opposite [18,35]. Also, a number of studies concluded that females experience increased diagnostic delay [8,10,11,14-16,20,27,28,30,40,41]. However, a substantial number of studies made the opposite conclusion [5,21,23,25]. In addition, history of immigration or illegal residency seemed to be a risk factor in countries where this is actual [8,15,17,22,38,39,42].

#### Socioeconomic factors

Thirteen studies found low income and poverty to be a risk factor for diagnostic delay [3,7,17,23,24,31,34,38,39,43-46]. In a range of studies, low educational level and/or low awareness and knowledge about TB was listed as a risk factor for diagnostic delay [5,10,16-18,21,23,25,27-29,34,37,47,48]. Only one, from France [13], determined the opposite, finding that low educational level was linked to immigration and socially deprived groups where the health authorities focused on TB.

Other socioeconomic risk factors identified by 1–3 studies included being married [5], being single [18,26], large family size [30], being a farmer [5], being white (vs. aboriginal) [20], being a Muslim [18], belonging to an indigenous group [13], and not having insurance [13].

#### Beliefs and attitudes

Beliefs about TB (TB is incurable, caused by evil spirits, etc.) [8,14,27], stigma [27], and self-treatment [6,36,42] were identified as risk factors in 1–3 studies.

#### The vicious circle of repeated visits at the same level

A majority of the studies identified as the direct or underlying problem a vicious circle of repeated consultations with a multitude of healthcare providers without a correct diagnosis. Several papers list multiple visits at the same level, while others focus on multiple visits to the same physician. Three groups of healthcare providers were particularly identified as sources of this vicious circle: primary-level government health posts, who have limited diagnostic facilities and poorly trained personnel [5,6,9-11,23,26,32-34]; private practitioners with low awareness of TB [9-11,14,15,19,21,23,25,26,29,32]; and unqualified vendors, quacks, and traditional practitioners [9,10,14,26-29,32,36,37] In Burkina Faso for a patient seeking a health post or a private practitioner, the progression towards specialized services was poor; patients had repeated consultations at the same level, such that more than 94% of patients underwent repeated courses of nonspecific antibiotics [49]. In Ghana, the health personnel at government health posts have poor training in diagnosing TB, and the specialized services of the NTP are overcentralized [11]. In a study in Malaysia, only 11% of patients received their diagnosis after the first consultation, and 45% received their first diagnosis after the third consultation [21]. Another study from Malaysia similarly indicates low awareness of the private practitioner as a key problem [36].

## Discussion

The studies had different definitions of a range of variables, for example, debut of symptoms, first appropriate health care provider, time to diagnosis, and start of treatment, and they were not directly comparable or suited for a meta-analysis. Rather than excluding studies that failed to meet strict scientific criteria (like in a meta-analysis), we tried to extract the "solid findings" from all of them to arrive on a more global understanding of diagnostic delay in TB. Our detailed analysis demonstrates that the interacting factors affecting patients' health-seeking behavior and the availability of TB treatment can be categorized as involving either the patient or healthcare. Most factors influence both patient and health care delay, but some factors were more closely related to patient delay: alcohol or substance abuse, poverty, low access to health care facilities, rural residence, old age, belonging to an indigenous group and incomprehensive attitudes, beliefs and knowledge about TB. Other factors were evidently more linked to health care delay: coexistence of chronic cough and/or other lung diseases, having extrapulmonary or negative sputum smear TB, less severe and indifferent symptoms or absence of haemoptysis, poor health care infrastructure and seeking traditional and private practitioners first. As patients continue to go untreated, absent isolation, both components equally contribute to the infectious pool.

Our analysis revealed that the vicious circle of repeated visits at the same level is the core problem of diagnostic delay in most high endemic countries. TB is a rare disease, and more than 95% of patients with chronic cough seeking treatment at the level of primary healthcare do not have TB [43]. The delay in diagnosis on the part of health providers does not necessarily reflect inferior performance, but instead a lack of effective diagnostic tools and follow-up routines. Correct diagnosis requires both good training and available diagnostic facilities. The number of repetitions of this vicious cycle proved to be highly dependent on both the patient's beliefs and awareness of TB and the category of healthcare provider. In most countries, private practitioners are consulted first, and this pattern is significantly associated with a longer diagnostic delay. This pattern is also recognized as a source of bottleneck in low endemic countries. A New Zealand study observed that the first consulting doctor often did not perform an X-ray, ask about previous TB, or obtain a sputum smear – although the patients had classical symptoms [44]. The same observations were make by Ward et al in Australia [20]. Cough was treated symptomatically, and CXR were misdiagnosed.

A range of studies have shown that selection of a traditional practitioner for the first visit is associated with a prolonged delay in diagnosis [9,10,14,26-29,32,36,37]. In Yimer et al's study from Ethiopia, patients who first visited a qualified medical provider experienced a 21-day delay before initiation of treatment. Patients who first visited a traditional healthcare provider waited 15 days before first seeking healthcare, but the period from first visit to initiation of TB treatment was 4-fold greater [6]. These findings are supported by studies of The Gambia [32], Tanzania [29], and Penang [21].

Many studies describe a bottleneck in reaching the local unit of the NTP. Multiple studies observed poor access to the NTP as one of the main factors in delayed diagnosis [6,8,10,14,18,23,25,27-30,34,42,47,48,50]. In many high endemic countries, it appears crucial that a unit of the NTP is within a 1-day walking distance, as many patients have limited access to motorized vehicles. In addition to geographic distance, the studies identified several other barriers. One of the most important barriers is stigma [27]. Many patients were highly reluctant to visit the NTP facility, because it would mean disclosing to the public that they had TB. Even worse, in many countries, TB is so closely linked to HIV that patients fear they are revealing HIV status to their neighbors [37]. Also, many of the NTP personnel are unfriendly. Many patients feel deprived of both privacy and dignity. The widespread introduction of DOTS also means that patients must visit daily so that their consumption of medication can be directly observed; this is perceived as humiliating, time consuming, and a threat of a substantial loss of income [6,51,52]. A marginal farmer or a day laborer will often have to choose between treatment and placing food on the table for his family. Most of the studies from developing countries demonstrated a significant link between delayed diagnosis and poverty [7,20,21,27,28,34,37,40,47,48].

Most of the studies in our review also demonstrate the nonspecific nature of symptoms and the disease's first natural history as a core problem [15,18,23,31,50,53]. In the Gambia, TB is often misdiagnosed as malaria or viral infections [32].

Several studies also highlight the problem of self-treatment [6,36,42]. In the study in Pakistan, more than 50% of patients practiced self-treatment, and 42% first searched a pharmacy for their symptoms[27]. Many studies link this to the problem of stigma, in fact the unhappy triad of incomprehensive beliefs, low awareness, and stigma.

## Conclusion

Our analysis is consistent with the findings of the WHO Eastern Mediterranean Region study. They concluded: "The private sector was the first choice for more than two-thirds of the patients. The main determinants of delay were: socio-demographic; economic; stigma; time to reach the health facility; seeking care from non-specialized individuals; and visiting more than one health care provider before diagnosis [3]." The core problem in delay of diagnosis and treatment seemed to be a vicious cycle of repeated visits at the same healthcare level, resulting in nonspecific antibiotic treatment and failure to access specialized TB services. Once generation of a specific diagnosis was in reach, TB treatment was initiated within a reasonable period of time.

## Competing interests

The author(s) declare that they have no competing interests.

## Authors' contributions

DGS, SY and GAB all contributed to develop the review protocol, DGS and SY performed data collection and analysis. DGS wrote the manuscript, and all the three authors edited and approved it.

## Pre-publication history

The pre-publication history for this paper can be accessed here:


